# Identification of human leukemia antigen A*0201-restricted epitopes derived from epidermal growth factor pathway substrate number 8

**DOI:** 10.3892/mmr.2015.3673

**Published:** 2015-04-23

**Authors:** BAISHAN TANG, WEIJUN ZHOU, JINGWEN DU, YANJIE HE, YUHUA LI

**Affiliations:** Department of Hematology, Zhujiang Hospital, Southern Medical University, Guangzhou, Guangdong 510280, P.R. China

**Keywords:** cancer immunotherapy, tumor-associated antigen, epidermal growth factor receptor pathway substrate 8, cytotoxic T lymphocyte, T-cell epitope

## Abstract

T-cell-mediated immunotherapy of hematological malignancies requires selection of targeted tumor-associated antigens and T-cell epitopes contained in these tumor proteins. Epidermal growth factor receptor pathway substrate 8 (EPS8), whose function is pivotal for tumor proliferation, progression and metastasis, has been found to be overexpressed in most human tumor types, while its expression in normal tissue is low. The aim of the present study was to identify human leukemia antigen (HLA)-A*0201-restricted epitopes of EPS8 by using a reverse immunology approach. To achieve this, computer algorithms were used to predict HLA-A*0201 molecular binding, proteasome cleavage patterns as well as translocation of transporters associated with antigen processing. Candidate peptides were experimentally validated by T2 binding affinity assay and brefeldin-A decay assay. The functional avidity of peptide-specific cytotoxic T lymphocytes (CTLs) induced from peripheral blood mononuclear cells of healthy volunteers were evaluated by using an enzyme-linked immunosorbent spot assay and a cytotoxicity assay. Four peptides, designated as P455, P92, P276 and P360, had high affinity and stability of binding towards the HLA-A*0201 molecule, and specific CTLs induced by them significantly responded to the corresponding peptides and secreted IFN-γ. At the same time, the CTLs were able to specifically lyse EPS8-expressing cell lines in an HLA-A*0201-restricted manner. The present study demon-strated that P455, P92, P276 and P360 were CTL epitopes of EPS8, and were able to be used for epitope-defined adoptive T-cell transfer and multi-epitope-based vaccine design.

## Introduction

Even though chemotherapy, radiotherapy and hematopoietic stem cell transplantation (HSCT) have been used to treat hematological malignancies, several of these are still considered incurable. Harnessing the host immune system towards the targeting of cancer cells for their destruction has been shown to be effective (1), and therapeutic vaccines against leukemia-associated antigens (LAAs) are among the approaches to trigger tumor-specific immune responses. Identification of LAA and its cytotoxic T lymphocytes (CTL) epitopes are of major importance for the development of tumor antigen-specific therapeutic and prophylactic vaccines. A number of T-cell epitopes from LAAs have been identified, and peptide vaccines targeting LAAs based on their T-cell epitopes have been shown to be effective in clinical trials (2).

Epidermal growth factor receptor pathway substrate 8 (Eps8) contains 822 amino acids and is located in chromosome 12p12-p13 in humans. The EPS8 gene encoded for the expression of proteins with molecular weights of 97 and 68 kDa, referred to as the two EPS8 isoforms. As a signaling adapter, EPS8 regulates actin cytoskeleton dynamics and architecture (3) and participates in transduction of signals from Ras to Rac by activating the Rac-specific guanine nucleotide exchange factor activity in the form of a trimeric complex together with SOS1 and ABI1 (4). It involves in the regulation of processes including dendritic cell migration as well as cancer cell migration and invasion (5).

Strong evidence demonstrated that EPS8 had oncogenic potential, and its overexpression was reported in a range of human malignancies, including breast cancer (6), pituitary tumors (7), pancreatic cancer (8), cervical cancer (9), colon cancer (10), head and neck squamous cell carcinoma (11), esophageal cancers (12) and human gliomas (13), and its amplification is often associated with tumor progression, acquired drug resistance and poor prognosis (14). EPS8 upregulation is thus expected to be involved in human carcinogenesis, implying that this gene may be used as a target in tumor immunotherapy. A previous study by our group demonstrated an elevated expression of EPS8 in patients with acute myeloid leukemia (AML), its overexpression was inversely correlated with overall survival of patients (15), and EPS8 protein as a vaccine reagent induced a CTL response in a murine breast carcinoma model (16). From the aforementioned facts, it was speculated that this gene may be a prognostic marker and could be a target for immunotherapy of hematological malignancies.

To the best of our knowledge, no T-cell epitopes of EPS8 have been reported to date. The aim of the present study was to identify human leukemia antigen (HLA)-A*0201-restricted epitopes for EPS8. To achieve this aim, the expression of EPS8 in a range of tumor cell lines was detected using western blot analysis and reverse transcription quantitative polymerase chain reaction (RT-qPCR), and their phenotypes were detected using a direct immunofluorescence method. Furthermore, screening for the EPS8 protein sequence was performed using various algorithms to predict the peptide-binding ability to the HLA-A*0201 molecule and its proteasome cleavage sites at the C-terminus to identify T-cell epitopes. The findings were validated *in vitro* by peptide-binding affinity assay and brefeldin-A (BFA) decay assay. The functional avidity of candidate peptide-specific CTLs was evaluated using an enzyme-linked immunosorbent spot (ELISPOT) assay and a cytotoxicity assay. Four peptides which were CTL epitopes of EPS8 were identified, and which may be used in vaccine design and tumor immunotherapy.

## Materials and methods

### Cell lines

A lymphoblast cell line, designated as T2 (174 × CEM. T2), was purchased from the American Type Culture Collection (cat. no. CRL-1992™; Manassas, VA, USA). This cell line is transporter associated with antigen processing (TAP)-deficient and expresses HLA-A2. In the absence of exogenous antigen peptide load, major histocompatibility complex (MHC) class I expression levels on its surface are very low due to the poor stability of non-peptide-loaded MHC class I molecules. The human erythroleukemia cell line K562 (cat. no. TCHu191), the human acute monocytic leukemia cell line THP-1 (cat. no. TCHu 57), the colon cancer cell line SW480 (cat. no. TCHu172) and the human breast tumor cell line MCF-7 (cat. no. TCHu 74) were purchased from the Type Culture Collection of the Chinese Academy of Sciences (Shanghai, China). The human acute myelogenous leukemia cell line KG1a was provided by Tianjin Institute of Hematology (Tianjin, China). MCF-7 was cultured in Dulbecco’s modified Eagle’s medium (DMEM; Invitrogen Life Technologies, Grand Island, NY, USA), T2 was cultured in Iscove’s modlfied Dulbecco’s medium (IMDM; Invitrogen Life Technologies) containing 20% fetal bovine serum (FBS; GE Healthcare Life Sciences, Logan, UT, USA), and the other cell lines were all cultured in RPMI-1640 medium (Invitrogen Life Technologies) supplemented with 10% FBS, 2 mol/l L-glutamine, 100 IU/ml penicillin and 100 *µ*g/ml streptomycin (Biological Industries Israel Beit Haemek Ltd., Beit Haemek, Israel). Cells were maintained in a humidified 37*°*C incubator with 5% CO_2_.

### Reagents

Mouse anti-human HLA-A2 monoclonal antibody (clone number, BB7.2) conjugated with fluorescein isothiocyanate (FITC) was purchased from BioLegend (San Diego, CA, USA; cat. no. 343304), rabbit monoclonal anti-EPS8 antibody (clone number, EPR6112) was purchased from Abcam Inc. (Cambridge, MA, USA; cat. no. ab12488; 1:1,000 dilution), the secondary goat anti-rabbit immunoglobulin (Ig)G antibody was purchased from Southern Biotechnology Associates Inc. (Birmingham, AL, USA; cat. no. 4030-05; 1:2,000 dilution), mouse anti-GAPDH monoclonal antibody conjugated to horseradish peroxidase (HRP) was purchased from Kangcheng Bio-Tech (Shanghai, China; cat. no. KC 5G5; 1:1,000 dilution) and Immobilon western chemiluminescent HRP substrate was purchased from Merck Millipore (Billerica, MA, USA). Lymphocyte separation medium was purchased from Tianjin Haoyang Biological Manufacture Co., Ltd. (Tianjin, China), recombinant human interleukin (IL)-2 and IL-7 were purchased from Peprotech (Rocky Hill, NJ, USA), β2-microglubulin and BFA were purchased from Sigma-Aldrich (St. Louis, MO, USA) and phytohaemagglutinin-M (PHA) was purchased from Biological Industries Israel Beit Haemek Ltd (Kibbutz Beit Haemek, Israel). Cell lysates, proteinase inhibitor phenylmethanesulfonylfluoride (PMSF) and bicinchoninic acid (BCA) protein assay reagents were all purchased from Beyotime Institute of Biotechnology (Nanjing, China), the cytotoxic non-radioactive cytotoxicity assay kit was purchased from Promega Corp. (Madison, WI, USA), the IFN-γ ELISPOT kit was purchased from U-CyTech Biosciences (De Uithof, Utrecht, The Netherlands), TRIzol reagent and SYBR green qPCR super mix were purchased from Thermo Fisher Scientific Inc. (Waltham, MA, USA), and the primers specific for human EPS8 and GAPDH were synthesized by Sangon Biotech Co., Ltd (Shanghai, China).

### Epitope prediction

To identify the potential T-cell epitopes for EPS8, the HLA-A*0201 allele was screened, covering a wide range of populations (17). First, the protein sequence was screened for the best binding epitopes using the algorithms SYFPEITHI (http://www.syfpeithi.de/) and Bioinformatics and Molecular Analysis Section (BIMAS; http://www-bimas.cit.nih.gov/). The cut-off score was adjusted to >20 for SYFPEITHI and T_1/2_>100 for BIMAS, and peptides matching these two criteria and which were shared by the two algorithms were selected. Second, the NetChop algorithm (http://www.cbs.dtu.dk/services/NetChop/) was used to predict whether the selected peptides in the first step would be cleaved at the C-terminus. Third, protein sequences were analyzed with five different algorithms for HLA-A*0201, including immune epitope database (IEDB; http://www.iedb.org/), NetMHC (http://www.cbs.dtu.dk/services/NetMHC/), SVMHC (http://www.sbc.su.se/~pierre/svmhc/), EpiJen (http://www.ddgpharmfac.net/epijen/EpiJen/EpiJen.htm) and Rankpep (http://imed.med.ucm.es/Tools/rankpep.html). Peptides predicted by at least four algorithms were selected. The known HLA-A*0201-restricted carcinoembryonic antigen peptide-1 (CAP-1, YLSGANLNL) was used as a positive control peptide (18), while the HBcAg-derived HLA-A*2402-restricted peptide HBc117e125 (EYLVSFGVW) was used as a negative control peptide (19), and all peptides were synthesized using standard 9-fluorenylmethyl-oxycarbonyl chemistry by the Chinese Peptide Company (Hangzhou, China). They were of >95% purity as determined by reverse-phase chromatography and their identity was confirmed by mass spectrometry. The lyophilized peptide powder was dissolved according to the manufacturer’s instructions in ultrapure water or in dimeth-ylsulfoxide (BioLegend), diluted in phosphate-buffered saline (PBS; Invitrogen Life Technologies) to a final concentration of 1 mg/ml and stored in aliquots at −80*°*C until use.

### Peptide binding affinity assay

To evaluate the binding affinity of each candidate peptide to the HLA-A*0201 molecule, a classic T2 peptide-binding assay was performed as described by Dervillez *et al* (20) with certain modifications. T2 cells are TAP-deficient and HLA-A*0201-positive, but due to the poor stability of non-peptide-loaded HLA-A*0201 molecules, its HLA-A*0201 expression levels on the surface are low. Exogenous peptides are able to induce the accumulation of HLA-A*0201 molecules, and thus, upregulation of HLA-A*0201 molecules on T2 cells may be detected by florescent intensity exchange which reflects peptide binding ability to HLA-A*0201 molecules. Briefly, T2 cells were incubated overnight with peptides (final concentration, 100 *µ*M) in IMDM serum-free medium containing human β2-microglobulin (final concentration, 3 *µ*g/ml) in a humidified 26*°*C incubator with 5% CO_2_. Following 18 h, the temperature was raised to 37*°*C for 2 h. Cells were harvested by gently transferring into a sterile centrifuge tube and centrifuged at 200 × g for 5 min. Subsequently, cells were washed twice, firstly with serum-free IMDM and then with cell staining buffer (BioLegend; cat. no 420201). The cells were then stained directly with anti-HLA-A*0201 monoclonal antibody conjugated to FITC for 30 min at 4*°*C, and then analyzed using an Elite flow cytometer (Beckman Coulter, Miami, FL, USA). Three duplicate wells were set to each group and experiments were repeated three times; CAP-1 was used as a positive control, HBc117e125 was used as negative control, and T2 cells without any added peptide was used as a background control. The fluorescence index (FI) was calculated using the following formula: FI = [mean fluorescence intensity (MFI)_sampl_−MFI_background_] / MFI_background_, where MFI_background_ represents the value without peptide. FI>1.5 indicated that the peptide had a high affinity for HLA-A*0201 molecules, 1.0<FI<1.5 indicated that the peptide had moderate affinity for the HLA-A*0201 molecule, and 0.5<FI<1.0 indicated that the peptide had low affinity for the HLA-A*0201 molecule.

### BFA decay assay

A BFA decay assay was performed to evaluate peptide-HLA-A*0201 complex stability as described by Saini *et al* (21) with certain modifications. Briefly, T2 cells were seeded at 1×10^6^ per well in 24-well plates and cultured with either the candidate peptides or the control peptide (final concentration, 100 *µ*M) at 26*°*C overnight in serum-free IMDM medium containing β2-microglobulin (final concentration, 3 *µ*g/ml) to accumulate peptide-receptive class I molecules at the cell surface. Following 18 h of incubation, cells were washed and incubated with 10 *µ*g/ml BFA dissolved in serum-free IMDM for 1 h at 37*°*C. Cells were harvested by gently transferring into a sterile centrifuge tube and centrifuged at 200 × g for 5 min, then washed and resuspended. The cells were then stained with anti-HLA-A*0201 fluorescent monoclonal antibody and analyzed using flow cytometry. The calculated MFIs were used as values for the time-point 0. In another group, cells were treated similarly to those above; following washing three times, cells were re-suspended in IMDM medium in the presence of 0.5 *µ*g/ml BFA and incubated in a humidified 37*°*C incubator with 5% CO_2_. Cells were harvested by gently transferring into a sterile centrifuge tube at the indicated time points and centrifuged at 200 × g for 5 min, washed and stained with anti-HLA-A*0201 fluorescent antibody. The stability of each peptide bound to HLA-A2 was measured as the DC_50_-value, which was defined as an estimate of the time required for a 50%-reduction of the MFI-value recorded at time 0. The DC_50_-value was calculated according to the formula: MFI at indicated time points / MFI at time 0 × 100%. Three duplicate wells were set for each group or experiments were repeated three times, with CAP-1 used as control.

### Detection of EPS8 expression and phenotypic analysis of target cell lines

#### RT-qPCR

RT-qPCR was performed to detect the relative mRNA expression levels of EPS8 in T2, MCF-7, K562, SW480, KG1a, TF1a, Raji and THP-1 cell lines. Briefly, total RNA was extracted using TRIzol reagent, purity was tested according to the ratio of optical density (OD)_260_/OD_280_, and RNA integrity was confirmed by 1% agarose gel electrophoresis (Beyotime Institute of Biotechnology). The RNA was reverse-transcribed using iScript Select cDNA Synthesis kit (cat. no. 170-8896; Bio-Rad Laboratories, Inc., Hercules, CA, USA) in which random nonomer primers and oligo dT primers were included to synthesize cDNA. Purified total RNA was first heat denatured at 85*°*C for 5 min, then reaction systems were established, including RNA (1.0 *µ*g), Oligo(dT) (0.5 *µ*l), random primers (0.5 *µ*l), dNTP (10 nM), RNase inhibitor (0.5 *µ*l), buffer (5X, 4 *µ*l) and M-MLV (0.5 *µ*l) with a total volume of 20 *µ*l. The reaction conditions were as follows: 30*°*C, 10 min; 42*°*C, 60 min; 85*°*C, 10 min. The quantification of PCR products was accomplished using Platinum^®^SYBR^®^ Green qPCR SuperMix-UDG (cat. no. 11733–038; Invitrogen Life Technologies) The reaction systems included cDNA, sense and antisense primers of EPS8 and GAPDH, 2X SYBR green qPCR Super mix and dH_2_O. In each 20 *µ*l reaction system, 5 *µ*l of cDNA (1:20 dilution) was mixed with sense and antisense primers (0.5 *µ*l each) of EPS8 and GAPDH, 2X SYBR Green qPCR Super mix (10 *µ*l) and ddH_2_O (4 *µ*l). The reaction conditions were as follows: 50*°*C, 2 min; 95*°*C, 2 min; 95*°*C, 15 sec; 60*°*C, 32 sec, for 40 cycles. The relative quantitative expressions of EPS8 in each sample were evaluated by detecting the CT value exchange (ΔCT, ΔΔCT) using the 2^−ΔΔCt^ method. The quality of the cDNA was confirmed by PCR analysis of GAPDH. All RT-qPCRs using the ABI PRISM 7900 sequence detection system (Applied Biosystems, Foster City, CA, USA) were performed in triplicate. Gene-specific PCR primers used to amplify EPS8 and GAPDH were designed as follows: For EPS8, sense, 5′-GATGGAGGAAGTGCAAGATG-3′ and anti-sense, 5′-GACTGTAACCACGTCTTCACA-3′; for GAPDH, sense, 5′-GGGAAACTGTGGCGTGAT-3′ and antisense, 5′-GAGTGGGTGTCGCTGTTGA-3′.

#### Western blot analysis

The protein expression levels of EPS8 in the cell lines were detected using western blot analysis. Cells were maintained in the indicated culture media and used during their logarithmic growth phase. Cells were lysed and protein was extracted from cell lysates using RIPA lysis buffer at 4*°*C in the presence of proteinase inhibitor PMSF to protect protein from degradation. PMSF was dissolved in isopropyl alcohol and diluted to 100 mmol/l. In the protein extraction procedure, PMSF was added to the RIPA lysis buffer at a volume ratio of 1:100. Sample protein concentrations were determined using the BCA protein assay according to the manufacturer’s instructions prior to addition of sample loading buffer (Beyotime Institute of Biotechnology). The supernatant containing protein was mixed with protein loading buffer (5X) at a ratio of 4:1, and following boiling in 100*°*C for 5 min, samples were centrifuged and stored at −20*°*C until use. A total of 30 *µ*g of each sample was electrophoresed on a 10% SDS-polyacrylamide gel and transferred to a polyvinylidene difluoride (PVDF) membrane (Merck Millipore). Following blocking in 5% skimmed milk (Beyotime Institute of Biotechnology) for 1 h at room temperature, the membrane was incubated for 24 h at 4*°*C in 5% skimmed milk with added primary antibody to human EPS8 (dilution rate, 1:1,000). The membrane was then washed three times with 0.5 % Tris-buffered saline-Tween 20 (Beyotime Institute of Biotechnology) and incubated in 5% skimmed milk with added HRP-conjugated secondary antibody (dilution rate, 1:20,000). Following additional washing, the membrane was incubated in Immobilon western chemiluminescent HRP substrate consisting of luminol reagent and peroxide solution and captured on chemiluminescence-sensitive film. For each sample, GAPDH was used as a loading control (dilution rate, 1:10,000).

#### Phenotypic analysis of target cell lines

Briefly, cells were incubated in recommended medium containing FBS and antibiotics as described above. During their logarithmic growth phase, cells were harvested by centrifuging at 200 × g for 5 min and resuspended in Cell Staining buffer. Following centrifugation at 350 × g for 5 min, the supernatant was discarded and cells were incubated with 5 *µ*l of Human TruStain FcX™ (cat. no. 422301; BioLegend) per 100 *µ*l of cell suspension for 5–10 min at room temperature for Fc receptor blocking. Subsequently, cells were stained directly with HLA-A2 antibody conjugated with FITC (3–5 *µ*l) and incubated on ice for 15–20 min in the dark. Following washing twice with Cell Staining buffer by centrifugation at 350 × g for 5 min, cells were resuspended in 0.5 ml Cell Staining buffer and 5 *µ*l/million cells of 7-AAD Viability Staining solution (cat. no. 420403; BioLegend) were added to exclude dead cells. Cells were incubated on ice for 3–5 min in the dark and analyzed with a Flow Cytometer. The HLA-A phenotype of the target cells was detected as described by Imai *et al* (22).

#### Peptide-specific CTL induction

Peptides were used to immunize peripheral blood mononuclear cells (PBMCs) to induce peptide-specific CTLs as previously described (23–25). The experiment was approved by the Institutional Ethics Committee of Southern Medical University (Guangzhou, China) and informed consent was obtained from all 32 donors. HLA-A*0201 phenotypic analysis of donors was completed using PCR-sequence-based typing by BGI Tech (Shenzhen, China), and PBMCs were purified using lymphocyte separation medium. ≥2×10^6^ PBMCs obtained from two HLA-A2.1+ healthy donors were incubated with the predicted peptides (final concentration, 10 *µ*g/ml) in RPMI-1640 medium. On day three, 20 U/ml IL-2 and 10 ng/ml IL-7 were added. Half the volume of the medium was replaced with fresh medium containing IL-2 (final concentration, 20 U/ml) and IL-7 (final concentration, 10 ng/ml) every 2–3 days. On day seven, cells were harvested by gently pipetting and transferred into sterile centrifuge tubes and centrifuged at 128 × g for 10 min. Following centrifugation, cells were washed and re-suspended in fresh medium containing IL-2 and IL-7. The PBMCs were repeatedly stimulated with peptides every 7 days, the stimulated frequency was at least three in order to harvest a sufficient number of CTLs. At the same time, the antigen signal could be fully presented to CD8^+^ T-cells in PBMCs. After 3 weeks of stimulation, these cells were used for the cytotoxicity assay. The main cells in total PBMCs were CD8^+^ T-cells. Following three times of peptide stimulation, the CTLs were harvested and used for functional analysis. Whenever necessary, cells were divided.

#### IFN-γ ELISPOT assay

To further evaluate peptide immunogenicity, an IFN-γ ELISPOT assay was performed according to the manufacturer’s instructions. In brief, ~4×10^6^/ml/well (or more) PBMCs were stimulated with peptides at a final concentration of 10 *µ*g/ml. On day three, IL-2 (final concentration, 10 U/ml) was added to each well. On day five, half of the volume of medium was replaced with fresh medium containing IL-2 with a final concentration of 10 U/ml. On day seven, PBMCs were stimulated with peptides again as above. On day nine, the microliter plates were coated overnight at 4*°*C with anti-IFN-γ monoclonal antibody. On day 10, cells were harvested for experiments. Peptide at a final concentration of 10 *µ*g/ml was added to ~2×10^5^ PBMCs per well in 100 *µ*l X-VIVO 15 medium (Lonza Group Ltd., Auckland, New Zealand) and cultured at 37*°*C in a humidified incubator with 5% CO_2_. Following 20–26 h of incubation, contents of all wells were discarded, and following washing, streptavidin-HRP solution was added into each well, and plates were incubated for 1 hour at 37*°*C. The PVDF membrane was washed thoroughly, 3-amino-9 ethylcarbazole substrate solution was added into each well and plates were incubated for 25 min at room temperature in the dark. Plates were then washed with demineralized water and air-dried at room temperature. Following the completion of the experiments, spot-forming cells (SFCs) were counted by Dakota Biotechnology company (Shenzhen, China). For all experiments, PHA stimulation was used for the positive control, PBMCs without added peptide were used as the negative control, and serum-free X-VIVO 15 medium from (Hebei Lonzer Chemicals Co., Ltd., Handan, China) was used as the background control. Experiments for each group were performed in triplicate.

#### Cytotoxicity assay

The functional avidity of peptide-specific CTLs was evaluated using a lactate dehydrogenase (LDH) release assay. For this, the non-radioactive cytotoxicity assay kit was used to detect the lysis effects of CTLs on target cells. Briefly, the induced CTLs were used as effective cells, and MCF-7 cells (HLA-A*0201+, EPS8+), T2 cells pulsed with corresponding peptides, T2 cells pulsed with irrelevant peptide, T2 cells only, THP-1 cells (HLA-A*0201−, EPS8−) and K562 cells (HLA-A*0201−, EPS8+) were used as target cells. The ratio of effective cells to target cells was set to 100:1, a constant number of target cells (5,000 cells/well) was added to the effectors, and at the same time, controls were set according to the manufacturer’s instructions. Following centrifugation at 250 × g for 4 min, cells were incubated in a humidified 37*°*C incubator with 5% CO_2_ in 100 *µ*l phenol red-free culture medium containing 5% FBS for 4–6 h. Fourty-five minutes prior to supernatant harvest, lysis solution (10X) was added to target cell maximum LDH release control and cells were cultivated continually in the same condition. Subsequently, cells were centrifuged at 250 × g for 4 min, 50 *µ*l supernatant was transferred to the corresponding well of a flat-bottomed 96-well enzymatic assay plate for LDH measurement. Experiments were performed in three duplicate wells and repeated three times. The percent cytotoxicity was determined using the following formula: %Cytotoxicity = [Experimental−Effector spontaneous−Target spontaneous] / [Target maximum−Target spontaneous] ×100%.

#### Statistical analysis

SPSS 17.0 software (SPSS, Inc, Chicago, IL, USA) was used for statistical analysis. One-way analysis of variance was used for comparing differences between groups. P<0.05 was considered to indicate statistically significant differences.

## Results

### In silico analysis of EPS8 protein for potential candidate peptides

To identify potential CTL epitopes of EPS8, the two algorithms SYFPEITHI and BIMAS were used to predict the potential peptides with high binding ability to the HLA-A*0201 molecule, which was a limiting step, resulting in a small number of peptides, and four peptides shared by those two algorithms were selected. The algorithm NetChop was then used to predict whether the peptides would be cleaved at the C-terminus, and only peptides with cleavage sites at the C-terminus were selected. Finally, five different algorithms were used to further confirm the selected peptides. Four 9-mer peptides with starting positions in the EPS8 protein sequence at 455, 276, 360 and 92, were finally selected (Table I). The positive and negative control peptides are also listed in the table.

### In vitro analysis of peptide affinity and binding stability to the HLA-A*0201 molecule

**Figure 1:**
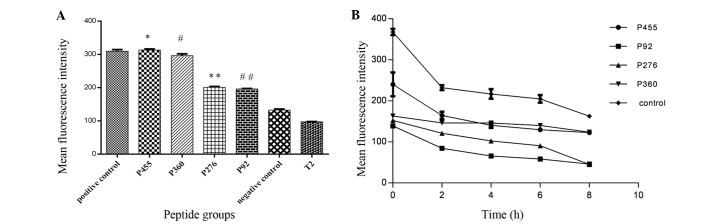
Affinity and stability of peptide binding to HLA-A*0201. (A) Affinity of candidate epitopes to the HLA-A*0201 molecule. ^*^P<0.01, P455 vs. P276, P360, P92, negative control and T2. ^#^P<0.01, P360 vs. P276, P92, negative control, positive control and T2. ^**^P<0.01, P276 vs. negative control, positive control and T2. ^##^P<0.01, P92 vs. negative control, positive control and T2. Values are expressed as the mean ± standard deviation (n=3). (B) Binding stability of candidate epitopes to HLA-A*0201 molecule. HLA, human leukemia antigen.

Evidence suggested that peptide affinity to MHC molecules is often correlated with its immunogenicity (26). Therefore, the TAP-deficient and HLA-A*0201-positive cell line T2 was used to detect peptide affinity for the HLA-A*0201 molecule. When exogenous peptides were added, the MFI of T2 cells increased significantly (Fig. 1A). Of the four candidate peptides, peptide 455 and 360 had the highest affinity to HLA-A*0201, with MFIs of 312.73±3.14 and 296.02±5.31, and FIs of 2.22 and 2.05, respectively. The FI values of the two peptides were almost as high as those of the positive control (FI=2.19). For peptides 276 and 92, the MFI was 200.77±2.60 and 195.96±1.35, and their FI was 1.07 and 1.02, respectively, indicating moderate affinity for the HLA-A*0201 molecule (P<0.001). The FI of the negative control was only 0.36 (Table I), and no weak-affinity peptides were found in the four candidate peptides according to the definition of peptide affinity for the HLA-A*0201 molecule.

In the BFA decay assay, with increasing time, the MFI of T2 cells in each group decreased (Fig. 1B), and the DC_50_-value was calculated to reflect the peptide/MHC complex stability; the longer the time, the more stable the complex. Complex stability of peptide 455/HLA-A*0201 and peptide 360/HLA-A*0201 was the highest, as their DC_50_-values were all >8 h, followed by peptide 276 with DC_50_<8 h, while the stability of peptide 92/MHC complex was lowest with a DC_50_ of 2–4 h (Table I).

### EPS8 expression detection and phenotypic analysis of target cell lines

**Figure 2:**
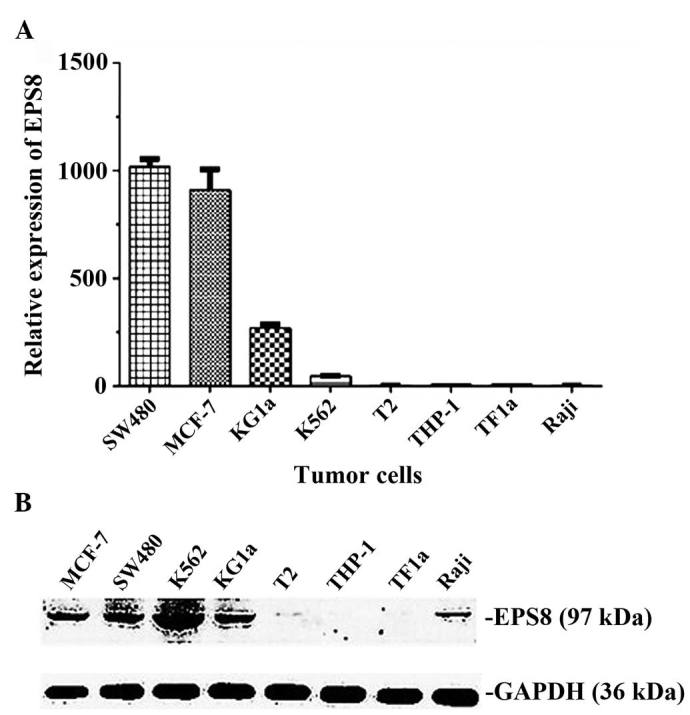
EPS8 expression of cell lines. (A) mRNA expression of EPS8, detected by reverse transcription quantitative polymerase chain reaction. Relative gene expression levels of EPS8 were compared using the 2^−ΔΔCt^ method, with GAPDH used as the internal control. Values are expressed as the mean ± standard deviation (n=3). (B) Protein expression of EPS8, detected by western blot analysis. GAPDH was used as the control. EPS8, epidermal growth factor receptor pathway substrate 8.

**Figure 3:**
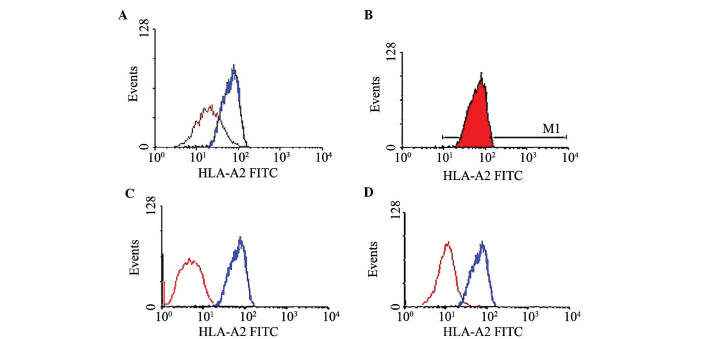
The HLA-A phenotype of target cells. (A) MCF-7, (B) T2, (C) THP-1 and (D) K562 cells. Cells were stained with HLA-A2-FITC monoclonal antibody and detected by flow cytometry. T2 cells were used as a positive control. The blue background lines represent T2 cells. MCF-7 cells were HLA-A*0201-positive, and THP-1 and K562 were HLA-A*0201-negative. FITC, fluorescein isothiocyanate; HLA, human leukemia antigen.

To identify candidate target cells, RT-qPCR was used to detect the mRNA expression of EPS8; furthermore, the protein expression of EPS8 was detected by western blot analysis. Among the eight cell lines, MCF-7, SW480, KG1a and K562 were EPS8-positive at the mRNA expression level (Fig. 2A) and the protein expression level (Fig. 2B). The HLA-A phenotype of the target cells was detected by direct immunofluorescence antibody staining and flow cytometric analysis. Of the eight cell lines, only MCF-7 and T2 were HLA-A*0201-positive (Fig. 3A and B).

### CTL epitopes of EPS8 stimulate PBMCs to express IFN-γ

**Figure 4:**
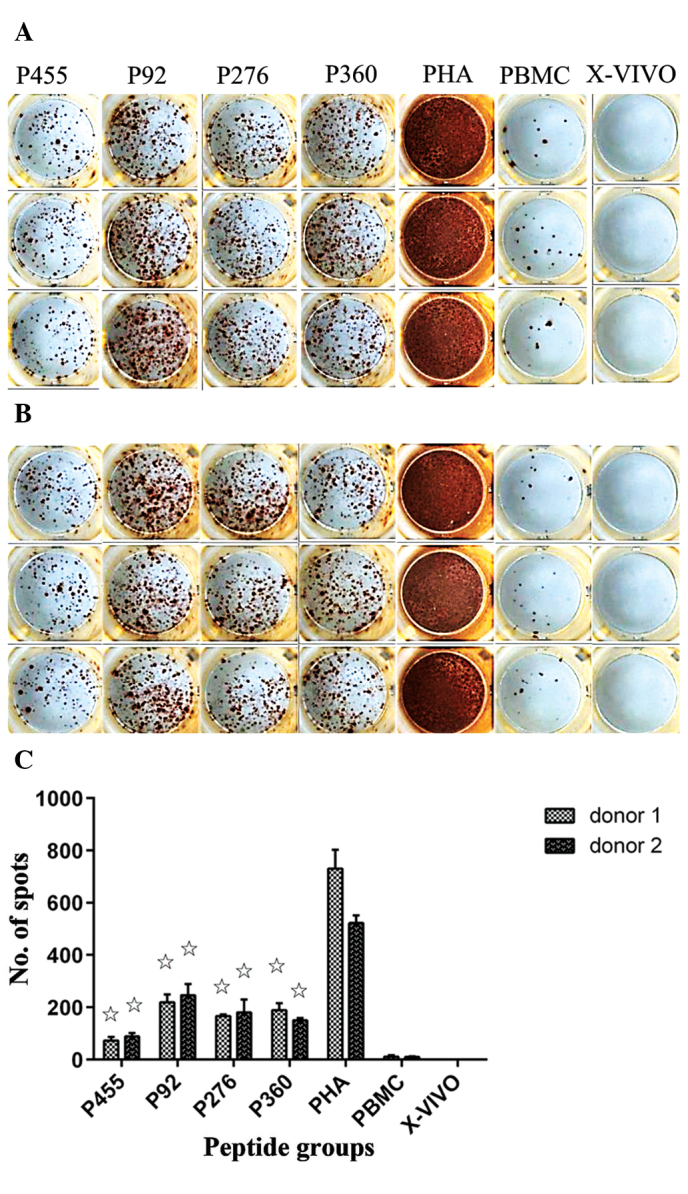
Interferon-γ secretion of specific CTLs in response to corresponding peptides. The number of SFCs was evaluated using an ELISPOT assay against four candidate epitopes of EPS8, with each experiment performed in triplicate with 2×10^5^ cells/well, and the average number of spots was calculated. PBMCs only served as a negative control. PHA stimulation served as a positive control. Bar graphs indicate the mean ± standard deviation. (A) Images of SFCs for volunteer 1. (B) Images of SFCs for volunteer 2. (C) Comparison of the number of SFCs of each candidate’s epitopes. Values are expressed as the mean ± standard deviation (n=3). ^✩^P<0.05 compared to negative control, X-VIVO medium. PBMC, peripheral blood mononuclear cell; PHA, phytohaemagglutinin-M; CTL, cytotoxic T lymphocyte; EPS8, epidermal growth factor receptor pathway substrate 8; SFC, spot forming cell.

To determine the immunogenicity of peptides, PBMCs were stimulated in a 10-day procedure as described in the Materials and methods section, followed by ELISPOT analysis. PBMCs separated from two HLA-A*0201-positive healthy volunteers were repeatedly stimulated with peptides, and the IFN-γ secretion of the stimulated PBMCs in response to the peptides was assessed for the two donors (Fig. 4). The PBMCs of the two donors responded positively to stimulation with all of the four predicted peptides, as indicated by an increased number of SFCs following stimulation. Of note, PBMCs responded strongly to stimulation with the relatively weak binder peptide 92 (218±30.4 peptide-specific spots/2×10^5^ cells), but elicited a response of lower intensity to strong binder peptide 455 (73.0±14.2 spots/2×10^5^ cells) (Fig. 4C) (P<0.001).

### The four peptide-specific CTLs specifically lyse EPS8 expression target cells in an HLA-A*0201-restricted manner

**Figure 5:**
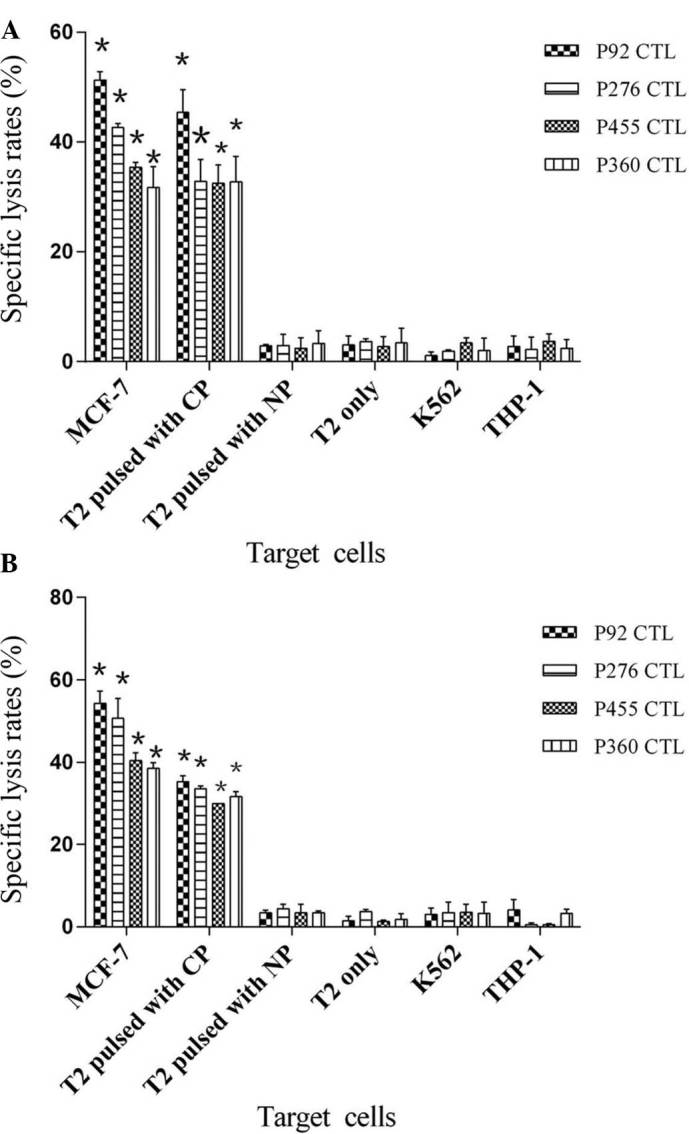
Functional characterization of candidate epitope-specific CTLs. LDH release assay using a ratio of effectors to targets of 100:1. (A) Results for volunteer 1; (B) Results for volunteer 2. Values are expressed as the mean ± standard deviation of LDH release. ^*^P<0.05 compared to negative control. CP, cognate peptides, NP, irrelevant peptide; CTL, cytotoxic T lymphocyte.

To characterize the functional avidity of peptide-specific CTLs, an LDH release assay was performed to evaluate their cytotoxic effects on MCF-7 and T2 cells pulsed with cognate peptides. As shown in Fig. 5, the four peptide-specific CTLs efficiently lysed MCF-7 as well as T2 cells pulsed with corresponding peptides but could almost not lyse T2 cells pulsed with irrelevant peptide, T2 only, K562 or THP-1 cells at an effector to target cell ratio of 100:1 (P<0.05). Of them, peptide 92-specific CTLs had the highest lysis rates of MCF-7 and T2 cells pulsed with the corresponding peptide, and the average lysis rate of MCF-7 cells was 51.28±1.54% (P=0.001; donor 1) (Fig. 5A) and 54.22±3.01% (P=0.001; donor 2) (Fig. 5B), respectively, while the lysis rate of T2 cells loaded with the corresponding peptide was 45.41±4.16% (P=0.023; donor 1) and 35.23±1.50%, (P=0.001; donor 2), respectively. The cytotoxic effects of peptide- induced CTLs on target cells were EPS8 expression-specific and restricted by the HLA-A*0201 molecule as the effectors only target HLA-A*0201 positive cells.

## Discussion

Concepts for treating hematologic malignancies by harnessing the immune system have been established due to defects of current treatment methods (27). HSCT is the only potential method to cure hematological malignancies, but relapse rates remain high, particularly in patients transplanted during relapse or in second or subsequent remission (28). Therefore, efforts are required to seek for novel avenues which are tumor-specific and have synergy with current treatment methods; from this viewpoint, therapeutic vaccines against LAAs are an optimal choice.

Mounting evidence demonstrated the existence of LAAs (29); as most LAAs are not muted, their immunogenicity is often poor; however, at the same time, the immunity evoked by current therapeutic vaccines targeting LAAs is far from optimal. Accordingly, isolating specific components that have immunogenicity and can induce a strong immune response are rational choices for vaccines. Therefore, the most important task in the discovery of cancer vaccines is to identify novel TAAs and their CTL epitopes in order to make additional therapeutic targets available which are closely associated with tumor incidence and progression. Reverse immunology has been commonly used to identify epitopes of LAAs (30).

The present study was the first, to the best of our knowledge, to report EPS8 as a potential TAA and identify its HLA-A*0201-restricted epitopes. EPS8 was reported to be expressed at elevated levels in numerous human malignancies and its overexpression has been reported to be sufficient to transform non-tumorigenic human cells into a tumorigenic phenotype (31). Its expression in normal tissue types is relatively low, and it has a pivotal role in tumorigenesis, tumor proliferation, invasion and metastasis; furthermore, the expression levels of EPS8 correlated with disease severity and overall survival of patients (32). Thus, EPS8 is a TAA and may be utilized as a target in immunotherapy of cancer as well as in hematological malignancies.

In the present study, a reverse immunology method was used to identify HLA-A*0201-restricted epitopes from EPS8. In the prediction phase, to appropriately select the T-cell epitopes contained in this protein, the focus was on three main events of the intracellular generation of peptides presented in HLA class I molecules; these HLA class I ligands are called CTL epitopes only when immunogenic (33). The first event is enzymatic digestion of protein by cytosolic peptidases, leading to the release of the epitope or epitope precursor in the protein. The second important event is required to prevent peptides from further cytosolic degradation via the TAP, only peptides that escape from cytosolic degradation and finally enter into endoplasmic reticulum can bind with HLA class I molecules. The third event is the assembly of peptides with HLA class I heavy and light chains in the ER for cell surface presentation (34–39). Peptides processed and presented through these three steps have the highest potential to be CTL epitopes. In the final step of the *in silico* analysis, five different popular algorithms were used to further confirm the selected peptides, leading to the final selection of four 9-mer peptides. It is worth mentioning that the aim of the present study was to identify dominant T-cell epitopes. For this, a combined prediction using SYFPEITHI and BIMAS was utilized, resulting in only four peptides, which significantly saved time by narrowing the validation scope. However, this does not indicate that there are only four CTL epitopes within the EPS8 protein sequence; due to stringent restrictions arising from the selection criteria, several potential subdominant epitopes may have been ignored. BIMAS and SYFPEITHY are based on the principle of binding motifs/quantitative matrices (39), and in spite of numerous novel algorithms that have emerged, they remain to be commonly used in current studies. The main reason for this may be the large dataset they comprise, which is important, as most predictions are dataset-driven-the bigger the dataset, the more accurate the prediction. One weakness of the motifs or quantitative matrices is the ignorance of the contribution of the overall peptide structure to binding. To overcome the limitations of motif-based predictions, more powerful methods based on tools including ANN, PSSM, SMM and SVMHC were used. It was reported that a sensitivity of 80% and a specificity of 80% can be achieved with ANN (40). Together with proteasomal cleavages and TAP translocation prediction, it was speculated that the anchor residues of selected peptides could chemically complement the main pockets of the peptide-binding groove of the HLA molecule.

The predicted peptides are not always true binders, and several false positive peptides may be selected (41); therefore, experiments are required to validate their immunogenicity. Peptide affinity to HLA molecules is a key event, and a correlation between HLA binding and immunogenicity is often observed (42). The eradication of tumor cells by T-cells requires high-affinity targeted peptide-MHC interactions, which lead to efficient cross-presentation of antigens. Their binding affinity to the HLA-A*0201 molecule was evaluated using the TAP-deficient cell line T2. When exogenous peptides were added, the HLA-A*0201 molecule on the surface of T2 accumulated, resulting in a change in fluorescent intensity, which changes in the MFI reflecting their binding affinity to the HLA-A*0201 molecule. Of the four peptides, peptide 455 and 360 had the highest affinity to the HLA-A*0201 molecule, while peptide 276 and 92 had a relatively low binding affinity to the HLA-A*0201 molecule. This meant that peptide 455 and 360 were most likely to be CTL epitopes.

However, certain peptides with high binding affinity to HLA class I molecules may be non-immunogenic (43), as they fail to form stable complexes with HLA class I molecules (44). Although peptide immunogenicity was found to correlate significantly with high affinity, the stability of peptide interaction with MHC-I correlated better with immunogenicity than affinity (45). Following the identification of the CTL epitopes, the present study assessed the peptide/MHC complex stability by using the BFA decay assay. BFA inhibits protein synthesis in cultured cells and reversibly inhibits the intracellular translocation of proteins to the cell surface for secretion or expression (46,47); therefore, the accumulated complex remained stable on the cells. When peptide-pulsed T2 cells were continuously cultured in the presence of BFA at 37*°*C, their MFI on the cells decreased, the peptide/HLA-A*0201 molecule complex dissociated naturally, and DC_50_-values indicated that the 455 and 360/HLA-A*0201 molecule complexes were more stable than those of peptide 276 and peptide 92. This meant that peptide 455 and 360 may be more immunogenic than peptide 276 and peptide 92.

High binding affinity and stable binding of peptides to MHC *in vitro* does not necessarily imply that these peptides can be naturally presented. To test the natural presentation of the candidate epitopes, the target cells were first screened by RT-qPCR and western blot to detect the EPS8 expression, and their HLA-A phenotype was detected by direct immune fluorescence antibody staining and flow cytometric analysis. Three tumor cell lines and T2 were selected as target cells. Most of epitope identification methods which have been used to date require the induction of peptide-specific CTLs, followed by their functional avidity evaluation; PBMCs separated from healthy donors are commonly used to evaluate the immunogenicity of a peptide (48). Two HLA-A*0201-positive donors were identified among healthy volunteers using an external high-resolution method. Peptide-specific CTLs were induced by immunizing the PBMCs of two healthy HLA-A*0201-positive donors through at least two rounds of weekly stimulations with peptides and in the presence of IL-2 and IL-7, the number of peptide-specific CTLs was sufficiently increased.

Following successful induction of specific CTLs, an IFN-γ ELISPOT assay was used to quantify the occurrence of T-lymphocyte cells secreting IFN-γ after stimulation with cognate peptide. The results demonstrated that the four peptide-specific CTLs all responded to the corresponding peptides and secreted IFN-γ; their effective SFCs met the criteria of the positive standard, with the number of SFCs of peptide 92 specific CTL being the highest. The results for the two donors were consistent, which meant that peptide 92 was more immunogenic than the other three peptides.

Finally, the recognition efficiency of peptide-specific CTLs to target cells was evaluated using a non-radioactive cytotoxicity assay, and in the two volunteers, all peptide-specific CTLs could lyse MCF-7 (HLA-A*0201+, EPS8+) and T2 cells pulsed with the same peptides, but could almost not lyse either HLA-A*0201-negative or EPS8-negative cells at the effectors to targets ratio of 100:1 (P<0.05). This indicated that their cytotoxicity was EPS8 expression-specific and in an HLA-A*0201-restricted fashion, the immunogenicity of peptide 92 was the strongest. Together with the ELISPOT results, it was demonstrated that the four predicted epitopes were naturally presented, and that they were CTL epitopes of EPS8.

In the present study it was observed that peptide binding affinity and peptide/HLA complex stability did not correlate well with their intensity of immunogenicity; for example, irrespective of its low affinity or complex stability with HLA-A*0201 molecule, the relatively weak binder peptide 92 stimulated PBMCs more strongly to secret the highest amount of IFN-γ than the strong binder peptide 455, which stimulated the least amount of IFN-γ secretion by the PBMCs. At the same time, compared to the mean lysis rates of CTLs induced by other peptides, the mean lysis rate of peptide 92-induced CTLs was the highest to MCF-7 and T2 pulsed with corresponding peptide. The reason of this may be based on the interaction dynamics of the T-cell receptor (TCR)-peptide/MHC complex, which is closely associated with T-cell activation and subsequently its differential fate (49). There is a physiological affinity range, which is optimal to activate T-cells, and a plateau of T-cell functional activity above a defined affinity threshold has been reported, suggesting that all clustered TCRs will be occupied above the defined threshold, and further increases in affinity do not contribute to T-cell functions; therefore, above a defined threshold, TCRs with higher avidity may not be at an advantage over lower-avidity TCRs in the exertion of cytotoxic function (50). Furthermore, negative feedback mechanisms, including programmed death 1 and lymphocyte activation gene-3 (CD223), would further curtail the potency of high avidity TCRs (51–53). The discrepancy observed in the present study may be ascribed to the peptide affinity of high-affinity binding peptides above the defined affinity threshold. Another reason may be the supra-optimal antigen dose. Evidence suggested that culturing of CTLs *in vitro* in the presence of high- or low- dose antigen leads to polarization of low and high-avidity responses, respectively (54,55). In regard to low- or intermediate-affinity peptides, the peptide dose used for CTL induction in the present study may have been supra-optimal for high-affinity peptides, with the optimal TCR stimulation peptide dose for maintenance of high-avidity T-cells *ex vivo* being 1 ng/ml and resulting in retained avidity, proliferation and ability to kill specific targets. By contrast, the supra-optimal TCR stimulation peptide dose (10 *µ*g/ml peptide) for high-avidity T-cell maintenance *ex vivo* resulted in reduced avidity and failure to kill tumor cells (56). The present study highlights the importance of optimal stimulation for the induction and maintenance of high-avidity CTL. Due to certain limitations, the present study did not further explore the best affinity to HLA molecules, which is in favor of peptide-specific CTL induction and maintenance.

Furthermore, the experiments of the present study had additional drawbacks: First, due to limitations to the amount of blood samples, the optimal cytokine concentration and their dosing intervals for best induction and maintenance of peptide-specific CTL were not explored. Second, background signals of target cells that lacked tumor antigens or restriction were observed, implying that non-specific killing effects still existed. The reason for this may be ascribed to the use of bulk CTL instead of CD8+ T-cell from the colon as effectors. Furthermore, CD8+ T cells were not sorted from PBMCs for CTL induction at the beginning and designated antigen-presenting cells such as dendritic cells (DCs) were not used; this may have resulted in inadequate antigen processing and presentation. Despite these drawbacks, from an economic and practical point of view, the methods used in the present study are an economical and rapid method for epitope validation. Furthermore, total PBMCs include a variety of cell types, including monocytes and DCs, which can be used as antigen-presenting cells (APC) (57).

It is worth mentioning that certain CTL epitopes identified *in vitro* may be less immunogenic *in vivo*, which may be due to inadequate antigen processing and presentation by APC (58). Therefore, a subsequent study by our group will test the immunogenicity of the epitopes *in vivo* using transgene mice.

In conclusion, the present study identified four HLA-A*0201-restricted, EPS8-derived epitopes, which are located at the starting positions of 92, 276, 360 and 455 in the EPS8 protein sequence. They had good binding affinity for the HLA-A*0201 molecule and their peptide/HLA-A*0201 complex stability was high. At the same time, these peptides promoted lymphocyte proliferation, and their specific CTLs responded to the corresponding peptides and secreted IFN-γ. They were also able to recognize and lyse ESP8-expressing target tumor cells in an HLA-A*0201-restricted manner, and they were naturally processed and presented on tumor cells. Among them, peptide 92 was the most promising as an antigenic epitope to target tumor cells due to its higher immunogenicity. Future studies will be required to investigate the clinical utility of the identified epitopes by using blood samples from patients with hematological malignancies.

## Figures and Tables

**Table I:** Characteristics of *in silico* predicted EPS8 CTL epitopes restricted to the HLA-A*0201 allele and control peptides.

Peptide^a^	Sequence	Score								FI^k^	DC_50_^l^
		SYFPEITHI^b^	BIMAS^c^	NetChop^d^	IEDB^e^	NetMHC^f^	Rankpep^g^/proteasome^h^	EpiJen^i^	SVMHC^j^		
455	QLAESVANV	30	655.88	+	0.7	SB	108.0/+	0.78	1.04	2.22	>8 h
92	KLLDAKGKV	26	480.07	+	2.1	WB	80.0/+	1.03	0.53	1.02	2–4 h
276	ILDDIEFFI	21	927.86	+	0.3	SB	68.0/+	0.45	0.34	1.07	6–8 h
360	FLFTPLNMV	28	2722.68	+	0.3	SB	−	0.32	1.06	2.05	>8 h
CAP 1^m^	YLSGANLNL	–	–	−	–	–	−	–	–	2.19	6–8 h
HBc117e125^n^	EYLVSFGVW	–	–	−	–	–	−	–	–	0.36	–

a Amino acid start position in the protein sequence;

b selection criteria: Score >20;

c selection criteria: T1/2>100;

d 3.1 server, prediction method: C-term, 3.0 and threshold, 0.5;

e prediction method: IEDB recommended, depicted in percentile rank, low percentile rank = good binders;

f version 3.4, 9-mer predictions using Artificial Neural Networks, strong binder threshold 50 nM, weak binder threshold score 500 nM, binding level (WB for weak binder, SB for strong binder);

g Prediction method: PSSMs, binding threshold set to 2%;

h Proteasome cleavage filter: on, cleavage model selection: One. + indicates that the peptide has a C-terminus predicted by the cleavage model used, − indicates that the peptide has no C terminus predicted by the cleavage model used;

i depicted in predicted IC_50_ value (nM), proteasome cut off: 0.1, TAP prediction cut off: 5, output cut off: 2%;

j prediction method: Support Vector Machines based on training data from the MHCPEP database. 0 is the default cut off used to discriminate binders from non binders;

k FI = [MFI_sample_ MFI_background_ ] / MFI_background_;

l DC_50_ was defined as an estimate of the time required for 50% reduction of the MFI value recorded at time 0. It was calculated using the formula: MFI at indicated time-points/MFI at time 0×100%;

m Positive control peptide; IEDB epitope ID: 74915, HLA-A*0201 restriction;

n Negative control peptide; IEDB epitope ID: 15061, HLA-A24 restriction. MFI, mean fluorescence intensity; MHCPEP, major histocompatibility complex peptides; PSSMs, position-specific scoring matrices; IEDB, immune epitope database; HLA, human leukemia antigen.
